# Reasons for not using smoking cessation aids

**DOI:** 10.1186/1471-2458-8-129

**Published:** 2008-04-22

**Authors:** Beatrice Gross, Leonie Brose, Anja Schumann, Sabina Ulbricht, Christian Meyer, Henry Völzke, Hans-Jürgen Rumpf, Ulrich John

**Affiliations:** 1 Institute for Epidemiology and Social Medicine, Ernst-Moritz-Arndt-University Greifswald, Germany; 2 Department of Psychology, Royal Holloway, University of London, UK; 3 Coordination Center for Clinical Trials, University Leipzig, Germany; 4 Institute of Community Medicine, Ernst-Moritz-Arndt-University Greifswald, Germany; 5 Department of Psychiatry and Psychotherapy, Research Group S:TEP (Substance Abuse: Treatment, Epidemiology and Prevention), University of Lübeck, Germany

## Abstract

**Background:**

Few smokers use effective smoking cessation aids (SCA) when trying to stop smoking. Little is known why available SCA are used insufficiently. We therefore investigated the reasons for not using SCA and examined related demographic, smoking behaviour, and motivational variables.

**Methods:**

Data were collected in two population-based studies testing smoking cessation interventions in north-eastern Germany. A total of 636 current smokers who had never used SCA and had attempted to quit or reduce smoking within the last 12 months were given a questionnaire to assess reasons for non-use. The questionnaire comprised two subscales: "Social and environmental barriers" and "SCA unnecessary."

**Results:**

The most endorsed reasons for non-use of SCA were the belief to be able to quit on one's own (55.2%), the belief that help is not necessary (40.1%), and the belief that smoking does not constitute a big problem in one's life (36.5%). One quarter of all smokers reported that smoking cessation aids are not helpful in quitting and that the aids cost too much. Smokers intending to quit agreed stronger to both subscales and smokers with lower education agreed stronger to the subscale "Social and environmental barriers".

**Conclusion:**

Main reasons for non-use of SCA are being overly self-confident and the perception that SCA are not helpful. Future interventions to increase the use of SCA should address these reasons in all smokers.

## Background

Smoking is the most important single cause of morbidity and mortality in industrialised countries [1]. Because smoking prevention will not affect tobacco-related mortality until the second half of the 21^st ^century, quitting by current smokers is the main way to achieve positive effects on mortality in a medium term [2]. Many smokers are interested in quitting. More than 70% of all smokers try to quit smoking at least once in their lifetime [3]. In the United States, 40% of all smokers reported that they had tried to quit within the last 12 months [4], and 70% reported that they wanted to quit [5]. In Germany, lower rates were found. Only 34% reported at least one quit attempt within the last 12 months, and 43% said that they wanted to quit [6].

To support smokers in their quit attempts, a wide range of smoking cessation aids (SCA) is available. SCA comprise methods and products to assist smokers in quitting through coping with psychological or physical aspects of nicotine dependence. Meta-analyses have shown that smoking cessation courses, nicotine replacement therapy, and bupropion can significantly increase success rates in quitting [7]. Even minimal interventions such as self-help materials have a small effect when compared with no intervention [8]. In contrast to the evidence about the efficacy of SCA, less than 23% of current smokers actually use SCA when trying to quit smoking according to general population studies in the US [9,10]. In Germany, 19% have used SCA in at least one quit attempt during their lifetime [11]. The question arises why smokers do not sufficiently use available SCA. Therefore, we assessed reasons for not using SCA in smokers who reported an unaided quit or reduction attempt.

A limited number of studies investigating the utilisation of SCA compared users and non-users. Those studies [10-12] found that the use of SCA is more likely among women, older persons, and persons with more than 12 years of education than among men, younger persons, and persons with less than 12 years of education. Smoking more cigarettes per day, having had more quit attempts in the past, and a higher Fagerström Test for Nicotine Dependence score were positively associated with SCA use. Furthermore, smokers utilising SCA are more frequently allocated to stages with enhanced motivation to stop smoking [11]. In the current study, we therefore examined whether demographic, smoking behaviour, and motivational variables predict the reasons for not using SCA among smokers reporting an unaided quit or reduction attempt. It has been shown that smoking more cigarettes per day, having a higher degree of nicotine dependence, and less quit attempts in the past are associated with less success in quitting smoking [13]. Furthermore, the efficacy of nicotine replacement therapy has been proven mainly for smokers smoking more than 10–15 cigarettes per day [14]. Thus, smokers with an unfavourable smoking behaviour (heavier smokers) are most likely to benefit from the use of SCA. Therefore we furthermore investigated whether the reasons for non-use vary between heavy and light smokers.

## Methods

### Study design

We used data from two smoking cessation intervention studies conducted in Western Pomerania, a rural region in north-eastern Germany. Both studies used a randomised controlled design and for both studies ethical approval has been obtained from the ethics committee of the physician chamber of Mecklenburg-Vorpommern at the University of Greifswald, Germany. One of these studies was conducted with a general population sample, the other one was conducted in general medical practices which in Germany also provide access to the general population. General population data revealed that more than 80% of the general population consult a physician at least once a year [15]. Both studies were designed to evaluate the effectiveness of brief motivational interventions. The interventions were based on the Transtheoretical Model of Behaviour Change [16]. This model provides a framework for describing, explaining, and influencing health behaviours such as tobacco smoking. Core elements are the five stages of change, three within current smokers to describe different levels of readiness to quit smoking and two within former smokers to distinguish short-term and long-term quitters, 10 processes of change, which refer to activities that people use to progress through the stages of change, decisional balance (the pros and cons of changing a behaviour), and self-efficacy (conceptualised as situation-specific confidence to refrain from smoking). This framework is used to tailor interventions to the individual needs of each smoker. The interventions were delivered in form of either individualised letters or personal counselling. Information about SCA was given to persons who planned to quit smoking within the next four weeks. The provision of medication or formal behavioural interventions was not part of the study protocol. In both studies, a baseline assessment and up to four follow-up assessments were carried out. All measures reported in the current paper were assessed in the baseline and the 6-month follow-up questionnaires and were identical in the population-based and the general practice study.

### Samples

#### Population-based sample

Subjects identified as current smokers in a general population health examination survey, the "Study of Health in Pomerania" [SHIP;[17]], were eligible as participants of the smoking intervention study. Inclusion criteria were (1) answering "Yes" to at least one of the following questions "Do you currently smoke cigarettes?", "Do you currently smoke cigars or cigarillos", "Are you currently a pipe smoker?" (2) being between 20 and 79 years and (3) providing written informed consent to participate in the study. Of 1,315 persons identified as smokers, 917 (69.7%) completed the baseline assessment, which was conducted from April 2002 to November 2002. The 6-month follow-up was completed by 760 persons (82.9%) [18]. Compared to non-responders participants had a higher educational level (OR = 1.894, CI: 1.279–2.805; OR = 1.572, CI: .945–2.614), but did not differ with regard to all other demographic and smoking behaviour variables described below. Both baseline and 6-month follow-up assessment were paper-pencil questionnaires mailed to the participant's home.

#### General practice patient sample

To yield a representative sample of patients attending the primary medical care system in the same region as the population-based study, a two-step sampling procedure was applied [19]. In the first step, a random sample was drawn from all practitioners registered for primary medical care in the given region. A total of 34, out of 39, practitioners took part in the study (participation rate 87.2%). In the second step, all consecutive patients visiting the practices were screened for smoking status by a research nurse for a period of three weeks. This recruitment phase lasted from April 2002 to September 2003. Of 2,016 identified smokers aged 18 to 70 years, 1,653 (82.0%) gave written informed consent to participate and 1,610 (79.9%) completed the baseline questionnaire, which was administered in the waiting room. At the 6-month follow-up, 1,234 patients (76.6%) were re-assessed via computer-assisted telephone interview or via paper-pencil questionnaire when a participant could not be reached by phone. Persons who completed the 6-month follow-up were older (OR = 1.025, CI: 1.015–1.034) and had a higher educational level (OR = 1.639, CI: 1.268–2.120; OR = 2.315, CI: 1.566–3.422) than non-responders. No differences have been found with respect to all other demographic and smoking behaviour variables described below.

#### Sample for analysis

The interventions were not designed to directly support the utilisation of SCA, thus we included participants allocated to the intervention as well as to the control condition. Taken both studies together, 1,994 participants completed the 6-month follow-up assessment. Of those, smokers smoking exclusively cigars, cigarillos or pipe, occasional smokers and smokers who stopped smoking during the study were excluded. The remaining 1,632 current daily cigarette smokers were asked: "Have you ever used one of the following SCA: nicotine replacement products (patch, spray, gum), bupropion, self-help materials (book, brochure, CD, videotape), smoking cessation courses, acupuncture or hypnosis treatment?". We included acupuncture and hypnosis, although effectiveness of these SCA has not been proven. However, it has been reported that they are used as often as other SCA [11,12]. Smokers stating never to have used any of those SCA were further asked to answer a questionnaire about the reasons for non-use of SCA in the past. Asking smokers why they have not utilised SCA requires that they have had an opportunity to use SCA. Therefore, only those 636 smokers additionally reporting at least one serious attempt to quit or to substantially reduce smoking in the past 12 months were included in the present analysis.

### Measurements/Assessments

A series of demographic, smoking behaviour, motivational, and health-related variables assessed at baseline was used as potential predictors of the reasons for not using SCA. Demographic measures included gender, age, marital status, and education, representing the three most common educational levels in Germany (<10, 10, >10 years at school). Smoking variables included cigarettes per day, age at onset of smoking (<16, 16–18, >18 years), number of quit attempts (0, 1, 2–5, >5) within the last 12 months and the Fagerström Test for Nicotine Dependence [FTND; [20]]. Smoking intensity was assessed using a German translation of the phrase: "Please indicate on a scale between 0 and 100 the intensity of your smoking.", with 0 being "not intensive at all" and 100 being "very intensive" [21]. We differentiated smokers not intending to quit within the next six months and smokers intending to quit within the next six months. Self-efficacy of behaviour change [22] was assessed using an instrument that asks subjects to indicate their confidence to refrain from smoking across nine tempting situations on a five-point Likert scale. Higher scores indicated stronger confidence. For assessment of the pros of non-smoking [22], the smokers indicated the importance of five statements about positive aspects of non-smoking on a five-point Likert scale. Higher scores indicated greater importance. General health was assessed with the EuroQol visual analogue scale [EQ-VAS; [23]], a self-rating scale ranging from 0 (worst imaginable health state) to 100 (best imaginable health state). We used a German version of the Five-Item Mental Health Inventory Screening Test [MHI; [24]] to assess affective mental health. In this version subjects indicated on a five-point rating scale (ranging from 1 "none of the time" to 5 "all of the time") the frequency of the occurrence of two anxiety-related and three mood disorder-related symptoms during the last month [25]. Higher sum scores indicated a more favourable mental health.

#### Questionnaire for not using SCA

At 6-month follow-up, a questionnaire was used to assess the reasons for not using SCA in persons who had never used any SCA and additionally reported an attempt to quit or reduce smoking within the last 12 months. The questionnaire was adapted from previous research on alcohol dependence and misuse [26,27]. We selected 10 items and modified them to refer to tobacco smoking. Four additional items were created to assess reasons for non-use of aids specifically for the field of tobacco smoking (Table 1). Subjects were asked to indicate their agreement on a five-point rating scale from 1 "not at all applicable" to 5 "very applicable". Exploratory factor analyses with oblique rotation revealed two factors that could be interpreted as the subscales "Social and environmental barriers" and "SCA unnecessary". Internal consistencies of the two scales were α = .80 and α = .57, respectively. Factor loadings ranged from .355 to .795 for subscale "Social and environmental barriers" and .250 to .539 for subscale "SCA unnecessary" (Table 1).

**Table 1:** Reasons for not using smoking cessation aids. Factor loadings, frequency distributions and means (SD) for individual items.

	Factor loading		Not at all applicable				Very applicable	
	Factor 1	Factor 2	1 (%)	2 (%)	3 (%)	4 (%)	5 (%)	M (SD)
Scale "Social and environmental barriers"								1.85 (0.83)
I did not know where to turn to in order to use the aids.	.408	.127	63.5	7.1	10.2	4.9	11.0	1.89 (1.41)
I was worried what other people would think about me.	.680	-.132	76.9	7.2	7.5	1.6	3.3	1.42 (0.95)
I thought it would take too much time and energy to use the aids.	.525	.062	54.6	11.3	15.6	6.1	9.0	2.00 (1.35)
I did not want to admit to myself that I needed the aids.	.355	.298	44.8	10.1	17.5	8.8	15.1	2.37 (1.51)
I was too proud to use the aids.	.650	.051	67.8	7.9	9.0	4.2	7.4	1.71 (1.26)
It was unpleasant or embarrassing to use the aids.	.795	-.086	72.3	6.4	7.5	3.9	6.0	1.59 (1.18)
I did not feel able to talk to others about my smoking.	.698	-.111	71.1	7.1	7.9	3.8	6.4	1.62 (1.20)
I thought the aids would cost too much.	.389	.222	51.9	8.8	11.8	7.1	16.5	2.25 (1.57)
I did not know that such aids existed.	.414	.096	66.2	6.4	7.1	3.5	12.1	1.83 (1.42)

Scale "SCA unnecessary"								2.92 (0.91)
I believed that the aids would not help me with my attempt to give up or reduce smoking.	.169	.371	37.3	11.2	23.4	8.8	15.7	2.53 (1.48)
I thought I would be able to quit or reduce smoking on my own.	-139	.518	17.9	7.9	15.7	16.0	39.2	3.52 (1.53)
I had the feeling that smoking did not constitute a big problem in my life.	.076	.250	21.9	11.0	26.9	16.7	19.8	3.02 (1.60)
I thought that I did not need these aids.	-.021	.539	29.7	7.9	18.9	13.2	26.9	3.00 (1.42)
I believed that nothing would help me in my attempt to give up or reduce smoking.	.199	.460	34.0	12.3	25.5	9.6	14.0	2.55 (1.43)

### Statistical analyses

All data analyses were conducted using SPSS 12.0 and STATA 9.0. Descriptive statistics were used to indicate the most important reasons for not using SCA. Percentage of endorsement of a reason for not using SCA was computed combining the two highest rating categories (rating 4 and 5), rejection was computed combining the two lowest rating categories (rating 1 and 2). Regression analyses were performed to predict the reasons for non-use of SCA. The two subscales "Social and environmental barriers" and "SCA unnecessary" were used as dependent variables. For both subscales, a mean score across all items was computed. Baseline assessments of demographic, smoking behaviour, motivational, and health-related variables were used as predictors. For rating scales (MHI, pros of non-smoking, self-efficacy) mean scores across all items (ranging from 1 to 5) were computed. Because data were collected as part of two smoking cessation intervention studies, preliminary analyses were conducted to ensure that the results were not biased by study membership (population-based vs. general practice patient sample) and study group membership (intervention vs. control condition). The scale "SCA unnecessary" differed significantly between the two studies (p < .05). Furthermore the use of SCA was significantly higher in the intervention than in the control condition (p = .001), thus we adjusted for study membership as well as study group membership in all regression analyses. In a first step, bivariate regression analyses were performed to identify possible predictors. In a second step, all univariate significant variables (p < .05) were included in a multivariable model. Because the scale "Social and environmental barriers" was highly skewed we dichotomised this scale using 1 as cut-off. One group consisted of persons who did not agree to any of the items (coded 0) and the other group consisted of persons who agreed to at least one of the items (coded 1). Thus for this scale logistic regression analyses were performed, whereas for the scale "SCA unnecessary" linear regressions were used. We defined smokers smoking at least 15 cigarettes per day as heavy smokers and smokers smoking less than 15 cigarettes per day as light smokers. Chi-square statistics were used to compare the percentage of endorsement of the reasons between heavy and light smokers. To examine predictors of the reasons for non-use in heavy smokers we reran the regression analyses excluding smokers smoking less than 15 cigarettes per day.

## Results

Among the 1,632 daily cigarette smokers, 361 (22.1%) had ever used SCA. Among the remaining 1,238 never-users (33 smokers did not provide any information about SCA use), 636 (51.4%) reported a quit or reduction attempt within the last 12 months and were therefore eligible for the present analyses. Those 636 participants were on average 38.25 (SD = 14.92) years old, 53.1% were male. Most of them (66.2%) had at least 10 years of school education and 72.5% were married or lived in a stable partnership. The number of cigarettes smoked per day ranged from 1 to 45 (mean 14.99; SD = 7.17), the mean FTND score was 2.84 (SD = 1.98) and 62.4% intended to quit within the next six months.

The most frequently endorsed reasons for not using SCA were "I thought I would be able to quit or reduce smoking on my own" (55.2%), "I thought that I did not need these aids" (40.1%) and "I had the feeling that smoking did not constitute a big problem in my life" (36.5%). One quarter agreed that SCA would not help them to stop or reduce smoking, that nothing would help in trying to stop or reduce smoking, that SCA would cost too much, and that they did not want to admit to themselves that they needed these aids (Table 1). The most frequently opposed reasons were "I was worried what other people would think about me" (84.1%), "It was unpleasant or embarrassing to use the aids" (78.7%) and "I did not feel able to talk to others about my smoking" (78.2%).

Univariate logistic regression analyses showed that men, smokers with less than 10 years of school education, smokers smoking more cigarettes per day, smokers with a higher FTND score, smokers smoking more intensive, and smokers intending to quit within the next six months had a higher agreement to the scale "Social and environmental barriers" (Table 2). The multivariable model including these variables revealed that a higher educational level decreased the odds while intention to quit within the next six months increased the odds for agreement to the scale. Linear regressions were performed to identify variables associated with the scale "SCA unnecessary". Intention to quit smoking within the next six months, and a worse mental health increased the agreement to this scale (Table 2). When both variables were entered simultaneously in a multivariable regression model, only intention to quit smoking remained significant.

**Table 2:** Logistic^a ^and linear^b ^regression to predict reasons for not using smoking cessation aids from demographic, smoking behaviour, motivational, and health-related variables

Prediction variables	Scale "Social and environmental barriers" OR (CI 95%)		Scale "SCA unnecessary" β-weight	
	univariate	multivariable	univariate	multivariable
demographic variables				
gender				
male^c^				
female	.559 (.371–.843)	.620 (.376–1.020)	-.019	-^d^
age	1.004 (.989–1.019)	-	-.065	-
educational level				
< 10 years^c^				
= 10 years	.382 (.219–.668)	.500 (.267–.936)	-.013	
> 10 years	.220 (.111–.434)	.297 (.139–.632)	-.012	-
marital status				
married/living with a partner^c^				
not married/not living with a partner	.783 (.486–1.262)	-	-.019	-
smoking behaviour variables				
cigarettes per day	1.048 (1.015–1.082)	1.015 (.969–1.063)	.071	-
FTND-Score^e^	1.274 (1.098–1.477)	1.206 (.992–1.466)	.041	-
age at onset				
< 16 years^c^				
16–18 years	1.219 (.758–1.959)		.012	
> 18 years	1.091 (.613–1.942)	-	-.070	-
quit attempts within the last 12 months				
0^c^				
1	1.379 (.780–2.438)		.011	
2–5	1.528 (.852–2.742)		.057	
>5	1.051 (.389–2.839)	-	.016	-
smoking intensity	1.012 (1.003–1.021)	1.001 (.990–1.013)	.076	-
motivational variables				
intention to quit within the next six months				
no^c^				
yes	1.583 (1.013–2.473)	2.008 (1.207–3.338)	.121**	.113**
pros of non-smoking	1.148 (.957–1.376)	-	.078	-
self-efficacy	.885 (.696–1.125)	-	.063	-
health-related variables				
mental health	.927 (.684–1.257)	-	-.083*	-.073
general health	.989 (.976–1.001)	-	.022	-

All analyses were adjusted for study membership (population-based vs. general practice patient sample) as well as study group membership (intervention vs. control condition).

^a ^Scale „Social and environmental barriers", due to substantial non-normality the scale was dichotomised using 0 = no agreement, 1 = at least minimal agreement

^b ^Scale "SCA unnecessary"

^c ^Reference category

^d ^Not sufficient for multivariable analysis

^e ^Because cigarettes per day were used as separate predictor, the FTND score was computed without cigarettes per day to avoid multi-collinearity

* p < .05; ** p < .01

Compared with light smokers (less than 15 cigarettes per day), a significantly higher percentage of heavy smokers (at least 15 cigarettes per day) endorsed the following reasons (Table 3): not wanting to admit to oneself to be in need of SCA, concerns that SCA would cost too much or would be unpleasant or embarrassing to use, the belief that nothing would help in the attempt to quit or reduce and that the use of SCA would take too much time and energy. Regression analyses in heavy smokers showed that smokers with more than 10 years of school education showed less agreement to the scale "Social and environmental barriers" than smokers with less than 10 years of school education (OR = .464, CI: .201–1.072; OR = .146, CI: .053–.397). Intending to quit smoking within the next six months was related to a stronger agreement to the scale "SCA unnecessary" in heavy smokers (β = .114, p = .042).

**Table 3:** Endorsement of reasons for not using smoking cessation aids for heavy (at least 15 cigarettes per day) and light (less than 15 cigarettes per day) smokers.

	heavy smokers (>= 15 CPD) endorsement		light smokers (<15 CPD) endorsement		χ^2^-statistics	
	yes % (N)	no % (N)	yes % (N)	no % (N)	χ^2^-value (df)	p-value
Scale "Social and environmental barriers"						
I did not know where to turn to in order to use the aids.	17.9 (28)	82.1 (267)	14.1 (38)	85.9 (232)	1.55 (1)	.213
I was worried what other people would think about me.	5.9 (19)	94.1 (305)	4.4 (12)	95.6 (258)	0.60 (1)	.439
I thought it would take too much time and energy to use the aids.	18.2 (59)	81.8 (266)	12.3 (33)	87.7 (236)	3.90 (1)	.048
I did not want to admit to myself that I needed the aids.	29.6 (96)	70.4 (228)	18.7 (50)	81.3 (218)	9.51 (1)	.002
I was too proud to use the aids.	12.0 (39)	88.0 (286)	11.2 (30)	88.8 (237)	0.08 (1)	.773
It was unpleasant or embarrassing to use the aids.	12.9 (42)	87.1 (283)	6.7 (18)	93.3 (249)	6.15 (1)	.013
I did not feel able to talk to others about my smoking.	11.4 (37)	88.6 (288)	9.0 (24)	91.0 (243)	0.91 (1)	.340
I thought the aids would cost too much.	28.8 (94)	71.2 (232)	18.1 (48)	81.9 (217)	9.20 (1)	.002
I did not know that such aids existed.	18.3 (59)	81.7 (264)	12.9 (34)	87.1 (229)	3.09 (1)	.079

Scale "SCA unnecessary"						
I believed that the aids would not help me with my attempt to give up or reduce smoking.	27.1 (88)	72.9 (237)	23.5 (63)	76.5 (205)	0.97 (1)	.321
I thought I would be able to quit or reduce smoking on my own.	57.5 (187)	42.5 (138)	55.9 (151)	44.1 (119)	0.16 (1)	.693
I had the feeling that smoking did not constitute a big problem in my life.	39.9 (129)	60.1 (194)	34.6 (93)	65.4 (176)	1.80 (1)	.179
I thought that I did not need these aids.	40.7 (133)	59.3 (194)	41.2 (110)	58.8 (157)	0.02 (1)	.897
I believed that nothing would help me in my attempt to give up or reduce smoking.	27.9 (90)	72.1 (233)	20.2 (53)	79.8 (210)	4.67 (1)	.031

## Discussion

The main finding is that the reasons for not using SCA can be seen in attitudes and beliefs of the smokers towards smoking cessation and SCA rather than in social and environmental barriers. Smokers who do not use SCA think to be able to quit or reduce smoking on their own. They do not perceive smoking as a problem and therefore do not believe to need help. That heavy and light smokers do not differ in their agreement to these reasons reflects a high confidence of both groups in their ability to quit without help but may also reflect an underestimation of the problems associated with smoking cessation particularly in heavy smokers. Furthermore smokers do not think that SCA are helpful. There seems to be a discrepancy between the scientifically proven effectiveness of SCA [7,8] and the individually perceived effectiveness in persons most in need of these aids, i.e., the smokers who failed in previous quit attempts. This is corroborated by a study showing that only 20% of smokers are convinced that SCA increase their chances in quitting [28]. In addition to these reasons, one important environmental barrier could be found. For a quarter of all smokers, the costs of SCA are a reason for non-use. The costs for e.g. nicotine replacement therapy are calculated not to extend the costs for an equivalent amount of cigarettes smoked before. But smokers may consider only the additional costs for SCA compared with no costs for quitting on one's own.

Although the most important reasons for non-use are the same in heavy and light smokers, there are differences between these groups. The expected costs as well as the belief that nothing would help in the attempt to quit or reduce are more relevant for heavy smokers. Furthermore the (social) aspects that using SCA means admitting to need help and the fear of embarrassment deter heavy smokers more strongly from SCA use than light smokers. So the belief that smoking cessation is something which has to be done on one's own seems to be more important for heavy smokers. This has to be kept in mind particularly when planning interventions for heavy smokers.

In interpreting these findings, the climate according to tobacco prevention and control must be taken into account. Germany is a country with low anti-smoking climate [29] and relatively high smoking prevalence, and the pressure to quit smoking is small. Smoking is widely accepted in the German society, which is reflected in a lower number of quit attempts and a lower motivation to quit than in the US [4,5] or other European countries [6,29]. Thus, fewer smokers have the experience of withdrawal symptoms and other problems impeding smoking cessation. Furthermore, in contrast to other countries such as the UK [30] and partly the US [31], in Germany costs for nicotine replacement therapy and bupropion are not covered by health insurance. Smoking cessation courses also require co-payments. This might explain why costs of SCA are given as a reason for non-use. It might also reflect that nicotine dependence is not sufficiently accepted by society as a disease for which free help should be offered. Our finding that 25 % do not use SCA because they do not want to admit needing help to themselves might reflect this feeling that smoking cessation should be done on one's own. These specific circumstances in Germany may compromise the generalisability of our findings across countries, because different smoking climates may influence the reasons for not using SCA.

Our findings suggest that interventions and campaigns aiming to increase the use of SCA, particularly in heavy smokers, and thus increase the number of successful quit attempts should address the following issues: First, if smokers are provided with fewer opportunities to smoke, the smokers' perception of their competences to quit smoking on their own might be adjusted. In this regard, smoking bans in public buildings or workplaces can be helpful. Second, higher perceived effectiveness of SCA is associated with more frequent use [28,32]. Public education campaigns should address this issue. One aspect in such campaigns might be that smokers as well as health care providers should be aware that SCA are no stand-alone solution. SCA assist in smoking cessation but require motivation to quit and behavioural efforts as prerequisites. Heavy smokers in particular refrain from using SCA because of the perceived ineffectiveness. This makes it even more important to clarify that the available SCA are effective in particular for heavy smokers. Third, costs are a barrier of SCA use. Studies have shown financial coverage of SCA to be associated with increased use, quit attempts, and successful quitting [33-35]. This implies that reducing the costs of SCA might have a positive effect on the use of SCA.

We found that smokers intending to quit smoking within the next six months showed stronger agreement to both subscales ("Social and environmental barriers" and "SCA unnecessary") than smokers not intending to quit. One interpretation might be that this reflects greater cognitive involvement of smokers who are further along in the cessation process. Those smokers have already made up their mind and decided not to use any kind of help. Therefore, their reasons for not using SCA are more elaborated. These results highlight the necessity to address SCA already in smokers who are not motivated to quit, before they have consolidated how they want to quit. This is in line with other studies which confirm that SCA should be offered to all smokers irrespective of their motivation to quit [36,37]. In heavy smokers the intention to quit was only related to the subscale "SCA unnecessary". This suggests that in heavy smokers not intending to quit the offer of SCA should be accompanied by a brief counselling to emphasise that smoking more cigarettes per day hampers smoking cessation [13] and that thus especially heavy smokers can profit from SCA such as nicotine replacement therapy [14]. Social and environmental barriers seem to be especially important for people with a lower educational level. This finding also stays significant when focussing exclusively on heavy smokers. Knowing that the use of SCA is more likely among people with higher education [12], additionally focussing on social aspects (e.g. talking to others) or environmental barriers (e.g. providing information about costs and places to turn to in order to get help) might be promising to increase the use of SCA in heavy smokers with lower education.

Our study has some limitations: (1) We assessed the reasons for non-use retrospectively in participants who unsuccessfully attempted to quit or reduce smoking. Thus cognitive processing resulting from the failure of the quit or reduction attempt and limited recall might have influenced our results. For example, participants who attempted and failed to quit might be likely to retrospectively report that SCA were unnecessary. Future studies should therefore assess reasons for non-use close to a given quit attempt, before the success or failure is obvious. (2) Our rationale was to focus on smokers reporting a quit or reduction attempt within the last 12 months. This constraint is founded in the wording of the questionnaire. Asking smokers why they have not utilised any SCA when trying to stop or reduce smoking assumes that they have had at least one opportunity to use any. Furthermore, assessing reasons related to an actual behaviour may be able to obtain more elaborated cognition. However, this means that our sample excluded smokers without a quit or reduction attempt. Therefore we were not able to investigate if and how general attitudes towards SCA influence whether a smoker tries to quit or reduce smoking. Future studies should therefore investigate whether those attitudes influence subsequent quit attempts. (3) We assessed reasons for non-use across a combination of all kinds of SCA. Reasons for non-use may differ between different kinds of SCA. For example, expenditure of time and money may vary between different SCA, which in turn may influence perceived reasons for non-use. In our study, we decided to assess the reasons for non-use globally for all kinds of SCA combined but not for single SCA, because in Germany information about SCA and the public awareness of the different kinds of SCA are low. Only 19% of current smokers have ever used SCA, with 2.8% reporting use of multiple SCA [11]. Thus differentiating reasons for non-use between SCA was not appropriate. (4) We adapted a questionnaire from alcohol dependence and misuse research to smoking cessation research. Although we added four smoking specific items, the reasons for non-use in the field of tobacco smoking might not have been covered completely. Psychometric development of a questionnaire with specific tobacco items is clearly desirable, but remains a task for future research. (5) Generalisation of our findings might be limited because our sample consisted of persons from two smoking cessation studies in Western Pomerania. However, both studies used population recruitment approaches, which assure the inclusion of a wide variety of smoking patterns, e.g. smokers not intending to quit within the next months. Because attrition was associated with educational level (population-based sample) and age as well as educational level (general practice patient sample), our findings might not apply to younger persons with a low educational level.

## Conclusion

Our findings suggest that the main reasons for non-use of SCA are being overly self-confident and the perception that SCA are not helpful. These reasons are modifiable in nature and thus indicate potential targets for future interventions to increase the use of SCA.

## Competing interests

All authors declare that they have no competing interests.

## Authors' contributions

As part of two comprehensive research projects "Population-based smoking interventions" (project 1) and "Proactive interventions for smoking cessation in General medical Practices" (project 2), the present study could only be accomplished in cooperation of a group of persons. UJ, CM and AS were responsible for conceptualising and managing project 1, CM, H-JR, SU and UJ for project 2. BG and LB had the idea for the current paper. LB performed initial analyses as part of her diploma thesis. BG analysed the data and wrote the manuscript. BG, UJ, H-JR and HV were significantly involved in data interpretation. All authors critically reviewed the manuscript and made important intellectual contributions. All authors have approved the final manuscript.

## Pre-publication history

The pre-publication history for this paper can be accessed here:


